# International consensus on (ICON) anaphylaxis

**DOI:** 10.1186/1939-4551-7-9

**Published:** 2014-05-30

**Authors:** F Estelle R Simons, Ledit RF Ardusso, M Beatrice Bilò, Victoria Cardona, Motohiro Ebisawa, Yehia M El-Gamal, Phil Lieberman, Richard F Lockey, Antonella Muraro, Graham Roberts, Mario Sanchez-Borges, Aziz Sheikh, Lynette P Shek, Dana V Wallace, Margitta Worm

**Affiliations:** 1 Department of Pediatrics & Child Health and Department of Immunology, Faculty of Medicine, University of Manitoba, Room FE125, 820 Sherbrook Street, Winnipeg, Manitoba, Canada, R3A 1R9; 2 Cátedra Neumonología, Alergia e Inmunología, Facultad de Ciencias Médicas, Universidad Nacional de Rosario, Rosario, Argentina; 3 Allergy Unit, Department of Internal Medicine, University Hospital, Ancona, Italy; 4 Allergy Section, Department of Internal Medicine, Hospital Universitari Vall d'Hebron, Barcelona, Spain; 5 Department of Allergy, National Hospital Organization, Sagamihara National Hospital, Clinical Research Center for Allergy & Rheumatology, Kanagawa, Japan; 6 Pediatric Allergy and Immunology Unit, Ain Shams University, Cairo, Egypt; 7 Allergy and Asthma Associates, Germantown, TN, USA; 8 University of South Florida Morsani College of Medicine, Tampa, FL, USA; 9 Department of Women and Child Health, Food Allergy Referral Centre, University of Padua, Padua, Italy; 10 University of Southampton Faculty of Medicine, Southampton, United Kingdom, David Hide Asthma and Allergy Research Centre, St. Mary’s Hospital, Isle of Wight, United Kingdom; 11 Centro Medico Docente La Trinidad, Caracas, Clinica El Avila, Caracas, Venezuela; 12 Center for Population Health Sciences, The University of Edinburgh, Edinburgh, United Kingdom and Division of General Internal Medicine and Primary Care, Brigham and Women’s Hospital/Harvard Medical School, Boston, MA, USA; 13 Department of Pediatrics, National University of Singapore, Singapore; 14 Nova Southeastern University, Fort Lauderdale, FL, USA; 15 Allergie-Centrum-Charité, Klinik fur Dermatologie und Allergologie, Charité, Universitatsmedizin, Berlin, Germany

**Keywords:** Anaphylaxis, Acute systemic allergic reaction, Epinephrine (adrenaline), H_1_-antihistamines, H_2_-antihistamines, Glucocorticoids, Food allergy, Venom allergy, Drug allergy, Exercise-induced anaphylaxis, Idiopathic anaphylaxis

## Abstract

ICON: Anaphylaxis provides a unique perspective on the principal evidence-based anaphylaxis guidelines developed and published independently from 2010 through 2014 by four allergy/immunology organizations. These guidelines concur with regard to the clinical features that indicate a likely diagnosis of anaphylaxis -- a life-threatening generalized or systemic allergic or hypersensitivity reaction.

They also concur about prompt initial treatment with intramuscular injection of epinephrine (adrenaline) in the mid-outer thigh, positioning the patient supine (semi-reclining if dyspneic or vomiting), calling for help, and when indicated, providing supplemental oxygen, intravenous fluid resuscitation and cardiopulmonary resuscitation, along with concomitant monitoring of vital signs and oxygenation. Additionally, they concur that H_1_-antihistamines, H_2_-antihistamines, and glucocorticoids are not initial medications of choice.

For self-management of patients at risk of anaphylaxis in community settings, they recommend carrying epinephrine auto-injectors and personalized emergency action plans, as well as follow-up with a physician (ideally an allergy/immunology specialist) to help prevent anaphylaxis recurrences.

ICON: Anaphylaxis describes unmet needs in anaphylaxis, noting that although epinephrine in 1 mg/mL ampules is available worldwide, other essentials, including supplemental oxygen, intravenous fluid resuscitation, and epinephrine auto-injectors are not universally available.

ICON: Anaphylaxis proposes a comprehensive international research agenda that calls for additional prospective studies of anaphylaxis epidemiology, patient risk factors and co-factors, triggers, clinical criteria for diagnosis, randomized controlled trials of therapeutic interventions, and measures to prevent anaphylaxis recurrences. It also calls for facilitation of global collaborations in anaphylaxis research.

In addition to confirming the alignment of major anaphylaxis guidelines, ICON: Anaphylaxis adds value by including summary tables and citing 130 key references. It is published as an information resource about anaphylaxis for worldwide use by healthcare professionals, academics, policy-makers, patients, caregivers, and the public.

## Introduction

The prevalence of allergic diseases is increasing worldwide, attributed in part to increased exposure to environmental allergens and pollutants; nevertheless, these diseases remain under-diagnosed and under-treated. Within the framework of the International Collaboration in Asthma, Allergy, and Immunology (iCAALL), the World Allergy Organization (WAO) and three member organizations of the WAO federation of allergy organizations (the American Academy of Allergy, Asthma and Immunology [AAAAI], the American College of Allergy, Asthma and Immunology [ACAAI], and the European Academy of Allergy and Clinical Immunology [EAACI]) have united to increase global awareness of allergic diseases and to promote their evidence-based management. In this collaborative outreach program, international consensus (ICON) documents are being published as resources to provide information about allergic diseases for physicians, other healthcare professionals, policy-makers, patients, caregivers, and the public [1].

This ICON: Anaphylaxis paper focuses on the principal anaphylaxis guidelines developed and published independently by the collaborating organizations from 2010 through 2014 [2-4], other anaphylaxis-relevant guidelines and publications from these organizations [5-22], and more than 100 additional key publications [23-124] that contribute to the evidence base for diagnosis, management, and prevention of anaphylaxis. In addition, it describes unmet global needs in the diagnosis and treatment of anaphylaxis in high-, mid-, and low-resource countries [125-130] and proposes an international agenda for anaphylaxis research.

## Methods

The ICON: Anaphylaxis author group was identified by the 2012-2013 WAO Board of Directors based on global representation and comprised mainly of allergy/immunology specialists who had contributed to previous national and international anaphylaxis publications.

The WAO Anaphylaxis Guidelines [2], the AAAAI/ACAAI Anaphylaxis Guidelines (Practice Parameters) [3] and the EAACI Anaphylaxis Guidelines [4] were circulated to all co-authors, who reviewed them, responded to four sequential calls for input about them sent by e-mail during 2013, and supplied additional relevant references. Each call for input consisted of a series of detailed questions about anaphylaxis that focused on definition, diagnosis, treatment in healthcare settings, and post-discharge management including prevention of recurrences. Co-authors were asked to review the collaborating organizations’ principal guidelines and identify areas where they concurred, areas where emphasis differed, and areas where little or no information was provided. They were also asked to provide their perspectives on unmet needs in anaphylaxis and propose topics for an international anaphylaxis research agenda. The co-author response rate was 100% to all but one call for input (86%).

The lead author collated the responses. Areas where the co-authors failed to agree were discussed by e-mail correspondence. The lead author then drafted the ICON: Anaphylaxis manuscript, which was developed further by internal reviews by the co-authors (with 100% participation) and revisions. This was followed by external review by the iCAALL Steering Group, final revisions, and submission for publication. The co-authors met in person to discuss the ICON: Anaphylaxis document on June 25, 2013 during the World Allergy & Asthma Congress in Milan.

## Overview of collaborating organizations’ principal anaphylaxis guidelines

An overview of the unique aspects of the principal anaphylaxis guidelines published by collaborating organizations is provided in Table 1[2-4]. Although they vary in some areas of emphasis and in style, length, and referencing, their recommendations on anaphylaxis are aligned with regard to clinical diagnosis, initial treatment, and prevention of recurrences.

**Table 1:** **Overview of collaborating organizations’ principal anaphylaxis guidelines**^**1**^

	**WAO Guidelines**	**AAAAI/ACAAI**^**2 **^**Guidelines**	**EAACI Guidelines**
**Year of publication**	2011	2010	2014
**Authors**	Simons FER et al (10 co-authors)	Lieberman P et al (15 contributors)	Muraro A et al (28 co-authors)
**Countries represented by authors**	Argentina, Canada, Egypt, Germany, Italy, Singapore United Kingdom, United States, Venezuela	United States	Canada, Denmark, France, Germany, Italy, Ireland, The Netherlands, Poland, Portugal, Spain, Switzerland, United Kingdom
**Methods**	consensus	consensus	consensus using AGREE II methodology
**Length (number of journal pages)**	7 page summary, 22 e-pages	4 page summary, 30 e-pages	20 pages
**References**	total: 150; 80% published 2006-13	total: 311; 13% published 2006-13	total: 133; 68% published 2006-13
**Tables, Figures, Boxes, Appendices**	9 tables; 5 figures (illustrations)	8 e-tables; 4 e-figures (algorithms)	2 figures (algorithms); 15 boxes; 4 online supplements
**Dissemination and uptake**	key figures on diagnosis and treatment translated^3^ and widely disseminated as posters, pocket cards and patient information cards	utilized by allergy/immunology specialists	published in 2014
**Unique aspects**	developed and funded by WAO; evidence-based; global perspective; key points highlighted using color illustrations; allergists’ role highlighted; anaphylaxis in limited-resource areas discussed; research agenda	developed and funded by AAAAI/ACAAI/Joint Council^2^; evidence-based; detailed information on triggers; specific information on refractory anaphylaxis; allergists’ role highlighted	developed and funded by EAACI; evidence-based; participants from many disciplines; allergists’ role highlighted; emphasis on practical aspects of long-term management; research agenda

^1^See text pages 2-3 for details, ^2^developed by the Joint Task Force on Practice Parameters, the AAAAI, the ACAAI, and the Joint Council of Allergy Asthma and Immunology, ^3^available in Arabic, English, French, German, Italian, Japanese, Korean (2014), Mandarin (2014), Polish, Portuguese, Russian, Spanish, and Turkish.

The widely disseminated WAO Anaphylaxis Guidelines [2], developed by the WAO Special Committee on Anaphylaxis, are supported by global assessments of essentials for anaphylaxis diagnosis and treatment [5-7] and yearly updates [8,9] of the evidence supporting the recommendations made in the guidelines. They provide a comprehensive, practical view of anaphylaxis. The text is closely linked with detailed illustrations that focus on patient risk factors, co-factors that amplify anaphylaxis, mechanisms, triggers, principles of clinical diagnosis, treatment, and post-discharge management. The illustrations on clinical diagnosis and prompt initial treatment have been translated into numerous languages and globally distributed as posters, pocket cards, and patient information cards.

The AAAAI/ACAAI Anaphylaxis Guidelines (Practice Parameters) [3] were developed by the Joint Task Force on Practice Parameters (representing the AAAAI, the ACAAI, and the Joint Council of Allergy Asthma and Immunology), which has a long history of publishing practice parameters on anaphylaxis [10-12] and related subjects [13-16]. These guidelines feature detailed descriptions of many anaphylaxis triggers and of idiopathic anaphylaxis. They describe clinical and laboratory diagnosis, initial treatment of anaphylaxis, treatment of refractory anaphylaxis, and post-discharge management. Many allergy/immunology specialists self-report adherence to these guidelines [17].

The EAACI 2014 Guidelines [4] were developed according to the Institute of Medicine Guidelines for Clinical Practice [18]. Co-authors included: allergy/immunology specialists, emergency medicine specialists, population health scientists, primary care physicians, and representatives from patient organizations. These guidelines are supported by previous guidelines [19] and relevant concurrent publications that include systematic reviews of the epidemiology of anaphylaxis [20] and management of anaphylaxis [21], and the EAACI Food Allergy Guidelines [22]. They emphasize the role of anaphylaxis education in long-term management and provide practical recommendations for training physicians, other healthcare professionals, patients, and caregivers [4].

## Comparative review of collaborating organizations’ principal anaphylaxis guidelines

Scrutiny of collaborating organizations’ guidelines reveals many areas of consensus, some areas where emphasis differs, and a few areas where minimal information is provided [2-4] (Table 2, 3, 4, and 5). Additional key publications representing global anaphylaxis research relevant to each area are cited as resources [5-124].

**Table 2:** **Essential information on anaphylaxis: summary of collaborating organizations’ principal anaphylaxis guidelines**^**1**^

	**WAO Guidelines**	**AAAAI/ACAAI Guidelines**	**EAACI Guidelines**
**Definition of anaphylaxis **	"a serious life-threatening generalized or systemic hypersensitivity reaction" and “a serious allergic reaction that is rapid in onset and might cause death"	"an acute life-threatening systemic reaction with varied mechanisms, clinical presentations, and severity that results from the sudden release of mediators from mast cells and basophils"	"a severe life-threatening generalized or systemic hypersensitivity reaction"
**Epidemiology**	not a major emphasis	not a major emphasis	summary of anaphylaxis epidemiology and clinical presentation: gaps in the evidence (Box 15)
**Patient risk factors and co-factors relevant to anaphylaxis**	describe vulnerability related to age, concomitant diseases (asthma, CVD, mastocytosis), concurrent medications (beta-blockers, ACE inhibitors); describe co-factors such as exercise, acute infection, emotional stress, premenstrual status, and ethanol or NSAID ingestion; Figure 1	describe concomitant diseases (asthma, CVD, mastocytosis), concurrent medications (beta-blockers, ACE inhibitors); mention premenstrual status as a co-factor	give examples of patient-specific factors, pre-existing conditions, medications and lifestyle factors; describe concomitant asthma in detail; Box 6
**Underlying mechanisms**	provide an overview of immunologic mechanisms (IgE-dependent and IgE-independent), non-immunologic (direct mast cell activation) and idiopathic anaphylaxis (no apparent trigger); Figure 2	describe immunologic mechanisms in the context of different anaphylaxis triggers; describe idiopathic anaphylaxis; Table E7	major focus on IgE-mediated anaphylaxis to food, insect venoms, and drugs; other mechanisms are mentioned
**Anaphylaxis triggers (causes, elicitors, or inducers)**	describe most triggers; state that the relative importance of specific triggers varies in different age groups and different global regions; Figure 2	describe many triggers in detail, with major emphasis on foods, venoms, drugs, biological agents, perioperative agents, radiocontrast media, latex, exercise, human seminal fluid, and idiopathic anaphylaxis; Table E5	overview of some triggers; describe food triggers in considerable detail; state that the importance of triggers varies with age and geography

^1^For details, see ICON: Anaphylaxis text pages 3-5 and references 2, 3 and 4, including the tables, figures, and boxes that are mentioned above in this Table.

ACE, angiotensin-converting enzyme; CVD, cardiovascular disease; NSAID, non-steroidal anti-inflammatory drug.

**Table 3:** **Diagnosis of anaphylaxis: summary of collaborating organizations' principal anaphylaxis guidelines**^**1**^

	**WAO Guidelines**	**AAAAI/ACAAI Guidelines**	**EAACI Guidelines**
**Symptoms and signs (typically within minutes to hours after exposure; multi-system; rapid progression)**	describe symptoms and signs in detail; Table 2	describe symptoms and signs; list frequency of symptoms and signs in different organ systems; Table E1	describe symptoms and signs; Figure 1
**Clinical criteria for diagnosis of anaphylaxis**	primary emphasis on clinical diagnosis; clinical criteria for diagnosis are listed and illustrated; Table 1, Figure 3	primary emphasis on clinical diagnosis: state “the history is the most important tool”; clinical criteria for diagnosis are listed; Figure E1	primary emphasis on clinical diagnosis: clinical criteria for diagnosis are listed; Box 4
**Laboratory tests for confirmation of the clinical diagnosis**	describe use of tryptase or histamine measurements and other tests in a supportive role; emphasize correct timing of blood samples; Table 3	describe use of tryptase or histamine measurements and other tests in a supportive role; emphasize correct timing of blood samples; Table E3	describe use of tryptase measurements in a supportive role
**Differential diagnosis**	comprehensive list provided; additional laboratory tests for ruling out other diagnoses are described; Table 4	comprehensive list provided; additional laboratory tests for ruling out other diagnoses are described; Tables E2, E3	comprehensive list provided, including detailed list of neuropsychiatric diseases; Box 5
**Diagnosis of anaphylaxis in special populations**	include reference ranges for vital signs in infants and children, and discuss relevant clinical and lab issues in infants, pregnant women and the elderly; Figure 1	include normal values for vital signs in infants and children; Table E4	describe patient-specific factors: examples include adolescence, advanced age, and gender; Box 6

^1^For details, see ICON: Anaphylaxis text page 5 and references 2, 3, and 4, including the tables, figures, and boxes from these references that are mentioned above in this Table.

**Table 4:** **Anaphylaxis treatment in healthcare settings: summary of collaborating organizations’ principal anaphylaxis guidelines**^**1**^

	**WAO Guidelines**	**AAAAI/ACAAI**^**2 **^**Guidelines**	**EAACI**^**3 **^**Guidelines**
**Prompt initial treatment of anaphylaxis**	have a protocol; remove the trigger, if relevant, assess rapidly, promptly and simultaneously call for help, inject epinephrine IM, repeat in 5-15 min, position the patient supine (semi-reclining if dyspneic or vomiting) with lower extremities elevated; Tables 5, 6, 7; Figure 4	epinephrine IM is the initial medication of choice; repeat in 5+ min; have a protocol; remove exposure to the trigger; position the patient supine (semi-reclining if dyspneic or vomiting) with lower extremities elevated; call for help; Figures E2, E3	1st-line treatment: ***inject*** epinephrine IM; repeat in 10 min; 2^nd^-line treatment: inhaled beta-2 agonists for wheezing; inhaled adrenaline for stridor; remove the trigger, call for help, position the patient appropriately, high-flow oxygen, fluid support (crystalloid); Boxes 7, 8, 15; online supplement; Figure 2
**Initial treatment (cont.)**	when indicated, give supplemental high-flow oxygen; IV fluids (crystalloid); start cardiopulmonary resuscitation with continuous chest compressions; H_1_- and H_2_-antihistamines, beta-2 agonists, and glucocorticoids are 2^nd^-line medications; Tables 5, 6, 7, 8; Figure 4	supplemental oxygen; IV fluid (crystalloid or colloid); cardiopulmonary resuscitation; H_1_- and H_2_-antihistamines, inhaled beta-2 agonists, and glucocorticoids are not initial medications of choice; Figures E2, E3	3^rd^-line interventions: H_1_- and H_2_-antihistamines, glucocorticoids; protocol for initial management includes cardiopulmonary resuscitation; systematic review of emergency management; Boxes 7, 15; online supplement; Figure 2
**Management of refractory anaphylaxis**	intubation; ventilation; IV vasopressors; glucagon; anticholinergic; transfer to hospital (preferably to an emergency medicine, critical care medicine, or anesthesiology) team for ventilatory and inotropic support; checklist of needed items; Table 6	vasopressors; dopamine; give vasopressin if epinephrine injections and volume expansion fail to alleviate hypotension; transfer to hospital; glucagon; atropine; methylene blue; includes checklist of supplies and equipment; Figures E2, E3	glucagon
**Observation and monitoring in healthcare settings**	observe for minimum 4 hrs; 8-10 hrs if respiratory or cardiovascular compromise; monitor BP, cardiac rate and function, respiratory status and oxygenation at frequent regular intervals, eg. 1-5 mins; continuous electronic monitoring if possible (essential if giving vasopressors); Table 5; Figure 4	individualize duration of observation; monitor BP and heart rate at frequent regular intervals (eg. 1 minute); continuous monitoring of BP, heart rate and function, and oxygenation, if possible; an example of a treatment record form for use in patients with anaphylaxis is provided; Figures E2, E4	minimum duration of observation 6-8 hrs for patients with respiratory symptoms and 12-24 hrs for those with hypotension or collapse; need for monitoring is highlighted; Box 7; online supplement; Figure 2

^1^For details, see ICON: Anaphylaxis text pages 5-8 and references 2, 3, and 4, including the tables, figures, boxes, and online supplemental materials from these references that are mentioned above in this Table.

^2^In the AAAAI/ACAAI Practice Parameters, one algorithm describes initial evaluation and management of a patient with a history of a previous episode of anaphylaxis and another algorithm describes treatment of an anaphylactic event in the outpatient setting.

^3^An evidence-based review of effectiveness of interventions for acute and long-term management is published separately.

BP, blood pressure; IM, intramuscular; IV, intravenous.

**Table 5:** **Anaphylaxis in community settings: summary of collaborating organizations’ principal anaphylaxis guidelines**^**1**^

	**WAO Guidelines**	**AAAAI/ACAAI Guidelines**	**EAACI Guidelines**
**Post-discharge management of patients treated for acute anaphylaxis**	prescribe epinephrine IM through auto-injector; anaphylaxis emergency action plan; medical ID stating triggers and co-morbidities; Table 9, Figure 5	prescribe epinephrine IM through auto-injector; anaphylaxis emergency action plan; medical ID; “an action plan is an important component of follow-up”; Figure E1	prescribe epinephrine IM through auto-injector; provide discharge advice sheet; provide specialist referral and contact information for patient support groups; Boxes 7, 9, 10, 11, 12, 13, 14, 15
**Investigations by allergy/ immunology specialists to confirm anaphylaxis triggers**	state the importance of the history of the episode; describe skin prick tests (intradermal tests needed for venom and drug-triggered anaphylaxis); investigations in idiopathic anaphylaxis; additional tests when needed to distinguish allergen sensitization from clinical risk in patients with food or drug allergy; Table 9, Figure 5	describe investigations in detail under different triggers; describe investigations in idiopathic anaphylaxis; details on skin testing with anesthetic agents; Table E8	state “validated in vivo and/or in vitro tests should be interpreted in the light of a detailed allergy history”; additional information re investigations to confirm food triggers is published separately in the EAACI Food Allergy Guidelines
**Prevention of anaphylaxis recurrences**	describe allergen avoidance (food, stinging insects, drugs, latex, etc.); immunotherapy with standardized insect venoms; and desensitization to drugs; mention food OIT^2^; describe pharmacologic prophylaxis of RCM anaphylaxis and idiopathic anaphylaxis; Table 9, Figure 5	describe specific avoidance measures under triggers; describe venom immunotherapy and desensitization to drugs; describe pharmacologic prophylaxis of RCM anaphylaxis and idiopathic anaphylaxis; Table E6; Figure E1	describe food avoidance; venom immunotherapy and desensitization to drugs; mention food OIT^2^; describe pharmacologic prophylaxis of anaphylaxis to RCM and snake anti-venom; Boxes 8, 9, 15; online supplement
**Anaphylaxis education and training**	outline principles of anaphylaxis education	provide relevant information under various triggers; give examples of print resources; Figure E1	major emphasis on all aspects of anaphylaxis training and adrenaline auto-injector prescription; Boxes 9, 10, 11, 12, 13, 14, 15; online supplement
**Follow-up with physician**	yearly review of EAI use, action plan, optimal management of co-morbid diseases, adjustment of concurrent medications as needed, allergen avoidance, and immune modulation	review discharge management, allergen avoidance, and immune modulation	major emphasis; include recommendations for training, management plan, and if relevant, help from nutritionists and psychologists; Boxes 9, 15; online supplement

^1^For details, see ICON: Anaphylaxis text pages 8-9 and references 2, 3, and 4, including the tables, figures, boxes, and online supplemental materials from these references that are mentioned above in this Table.

^2^Described but not recommended for clinical implementation at this time.

EAI, epinephrine auto-injector; ID, identification; IM, intramuscular; OIT, oral immunotherapy; RCM, radiocontrast media.

### Definition of anaphylaxis

In these guidelines, the independently developed definitions of anaphylaxis for clinical use by healthcare professionals all include the concepts of a serious, generalized or systemic, allergic or hypersensitivity reaction that can be life-threatening or fatal. Importantly, none of the definitions include the word “shock” [2-4] (Table 2). The correct term “anaphylaxis” is preferred to “anaphylactic shock” because shock is not necessarily present in patients with anaphylaxis [23-26]. The term “anaphylaxis” should also be used in preference to terms such as “allergic reaction”, “acute allergic reaction”, “systemic allergic reaction”, “acute IgE-mediated reaction”, “anaphylactoid reaction”, or “pseudo-anaphylaxis” [2-4].

### Epidemiology

None of the guidelines has a major focus in this area [2-4]; however, they all include important information about anaphylaxis epidemiology supported by relevant references [2-4,27-37] (Table 2).

### Patient-specific risk factors and co-factors relevant to anaphylaxis

The guidelines concur about the importance of patient-specific risk factors and co-factors in anaphylaxis [2-4]. The WAO Guidelines emphasize risk factors related to age (infancy, adolescence, advanced age), physiologic state (pregnancy), concomitant diseases including asthma, cardiovascular diseases (CVD), and mastocytosis, and concurrent medications such as beta-blockers and angiotensin-converting enzyme (ACE) inhibitors. The AAAAI/ACAAI Guidelines describe patient-specific risk factors such as asthma, CVD, and mastocytosis, and concurrent beta-blocker and ACE-inhibitor use. The EAACI Guidelines include a major focus on asthma as a patient risk factor (Table 2).

The guidelines also concur about the relevance of co-factors that amplify [2-4] anaphylaxis. The WAO Guidelines and EAACI Guidelines provide an overview of co-factors such as exercise, emotional stress, acute infection, fever, concomitant ingestion of ethanol or a non-steroidal anti-inflammatory drug (NSAID), disruption of routine, and perimenstrual status. The AAAAI/ACAAI Guidelines focus on exercise. The importance of risk factors [38-49] and amplifying co-factors [33,50,51] that potentially impact anaphylaxis is now widely acknowledged; indeed, co-factors are now reported to be relevant in 20-30% of anaphylactic episodes [33,50].

None of the guidelines describe the relationship between mast cell activation disorders (MCAD) and anaphylaxis, perhaps because the first international consensus document on classification of MCAD was only published in 2012 [45].

### Underlying mechanisms

The WAO Guidelines provide a brief overview of IgE-dependent and IgE-independent immunologic mechanisms and direct mast cell stimulation in anaphylaxis [2]. The AAAAI/ACAAI Guidelines describe immunologic mechanisms and direct mast cell activation in the context of different triggers [3]. The EAACI Guidelines focus mainly on IgE-mediated anaphylaxis [4] (Table 2).

None of the guidelines provide optimal information on IgG-mediated anaphylaxis in humans, an emerging area of investigation [52].

### Anaphylaxis triggers (Causes, Elicitors, or Inducers)

The WAO Guidelines describe triggers and idiopathic anaphylaxis concisely, and note that different triggers predominate in different age groups and different global regions [2]. The AAAAI/ACAAI Guidelines provide detailed information about many triggers including foods, stinging insect venoms, drugs, biological agents, perioperative agents, radiocontrast media, latex, subcutaneous allergen immunotherapy, and human seminal fluid; also about idiopathic anaphylaxis [3]. The EAACI Guidelines emphasize food triggers, provide information on stinging insect venom and drug triggers, and state that the relative importance of triggers varies with age and geography [4] (Table 2).

Anaphylaxis triggers such as food, venom, and drugs have been studied in Australia, Europe, North America, and beyond; for example, in Asia (China, Japan, Korea, Singapore, and Thailand), and South America (Argentina, Brazil, and Venezuela) [27-37,53-66]. In any region, the relative importance of triggers can change over time [54].

### Clinical diagnosis of anaphylaxis

Collaborating organizations’ guidelines concur about making the clinical diagnosis of anaphylaxis based on recognition of sudden onset of characteristic symptoms and signs within minutes to hours after exposure to a known or likely trigger [2-4]. They all list the clinical criteria for diagnosis of anaphylaxis that were developed as an instrument for rapid assessment of patients who present with a possible diagnosis of anaphylaxis [23] and are validated for use in medical settings and in epidemiologic studies [67,68] (Table 3).

The guidelines also concur that laboratory tests are not helpful in diagnosing anaphylaxis at the time of patient presentation [2-4]. Measurement of a biologic marker such as serum total tryptase takes hours and test results are not available on an emergency basis. Elevation in biologic marker levels correlates with anaphylaxis severity [24]; platelet-activating factor levels appear to correlate better than tryptase or histamine levels do [69]. Tryptase levels are elevated in only about 60% of adults with clinically confirmed anaphylaxis [70] and are seldom elevated in children with anaphylaxis or in food-induced anaphylaxis. The reference range for tryptase levels in infants has been established [71]. Tryptase levels (or levels of any other biologic markers) within the normal reference range do ***not*** rule out anaphylaxis. Lack of availability of biologic marker measurements is ***not*** a barrier to prompt clinical diagnosis of anaphylaxis [2-4].

In addition to the above, all the guidelines provide a perspective on the differential diagnosis of anaphylaxis [2-4] (Table 3).

### Prompt initial treatment of anaphylaxis

Importantly, collaborating organizations’ guidelines concur with regard to recommendations for prompt initial treatment of anaphylaxis with epinephrine (adrenaline) injected intramuscularly in the mid-outer thigh, and repeating the epinephrine dose after 5-15 minutes if the response to the first injection is not optimal [2-4] (Table 4).

The guidelines concur about the importance of preparedness to diagnose and treat anaphylaxis and about other measures such as rapid assessment of the patient, removing the trigger if possible, and calling for help. They all recommend positioning the patient supine (or semi-reclining in a position of comfort if dyspneic or vomiting) with elevation of the lower extremities [2-4].

The guidelines also concur that if indicated at any time, supplemental oxygen, intravenous (IV) fluid resuscitation with a crystalloid such as 0.9% (isotonic) saline, and cardiopulmonary resuscitation should be started without delay [2-4] and that H_1_-antihistamines, H_2_-antihistamines, and glucocorticoids are not initial medications of choice [72-75].

There are a few differences in emphasis among the guidelines with regard to initial treatment of anaphylaxis. The WAO Guidelines include the revised resuscitation guidelines recommendations for starting cardiopulmonary resuscitation with chest compressions before giving rescue breaths, allowing time for complete chest recoil, and minimizing interruptions for pulse checks [2,76]. The WAO Guidelines and AAAAI/ACAAI Guidelines describe epinephrine injection, calling for help, and positioning the patient appropriately as concurrent initial steps [2,3], while the EAACI Guidelines describe epinephrine injection as a first-line intervention and calling for help and positioning as second-line interventions [4]. The WAO Guidelines and the AAAAI/ACAAI Guidelines do not specifically recommend inhaled epinephrine for patients with stridor during anaphylaxis, although they note that an inhaled beta-2 agonist should be considered in patients with bronchospasm that persists despite epinephrine treatment [2,3]. The EAACI Guidelines recommend inhaled epinephrine (adrenaline) in patients with stridor and an inhaled beta-2 adrenergic agonist in patients with wheezing after, and in addition to, epinephrine injection [4] (Table 4).

None of the guidelines discuss epinephrine injection in high-risk patients after exposure to a relevant trigger but ***before*** symptoms develop and none discuss epinephrine administration to a patient diagnosed incorrectly with anaphylaxis, perhaps because there is little or no published information on either of these issues.

Epinephrine utilization in anaphylaxis remains an active area of research. The need for prompt epinephrine use to prevent escalation of mediator release in anaphylaxis has been confirmed in a new in vitro model [77]. Use of multiple epinephrine injections does not necessarily correlate with patient obesity [78]. Rates of epinephrine utilization as the initial medication for anaphylaxis in emergency departments are typically low [79]; however, they can be improved significantly with implementation of an anaphylaxis protocol [80,81].

### Management of anaphylaxis refractory to initial treatment

The guidelines differ in emphasis on refractory anaphylaxis treatment. The WAO Guidelines stress the importance of prompt initial treatment to prevent escalation of symptoms [2]. They suggest that if possible, patients with anaphylaxis refractory to epinephrine, supplemental oxygen, IV fluids, and second-line medications should be transferred to the care of a specialist team for ventilatory and inotropic support and continuous electronic monitoring [2]. The AAAAI/ACAAI Guidelines provide details about interventions for cardiopulmonary arrest, airway management, and IV administration of vasopressors including epinephrine, dopamine, and vasopressin [3]. The EAACI Guidelines include brief specific instructions about when to call for Intensive Care Unit support [4] (Table 4).

Studies relevant to refractory anaphylaxis treatment are of interest. A Cochrane review of randomized controlled trials (RCT) in more than 20,000 critically ill patients with distributive shock supports administration of crystalloids such as 0.9% saline, because administration of colloids such as albumin or hetastarch did not correlate with increased survival [82]. Methylene blue administration for vasoplegia in anaphylaxis refractory to epinephrine and IV fluid resuscitation is based on case reports and extrapolation from use in other forms of shock [83].

### Observation and monitoring in healthcare settings

The guidelines concur that patients with moderate or severe anaphylaxis, for example, those with moderate or severe respiratory or cardiovascular symptoms and signs should be observed and monitored for a longer duration (eg. at least 6-8 hours) than those with mild anaphylaxis. The WAO Guidelines note that duration of monitoring can also vary with patient age and co-morbidities, and with local conditions. The AAAAI and EAACI Guidelines provide additional information about biphasic anaphylaxis and protracted anaphylaxis [2-4] (Table 4).

In a prospective study in which mediator release in anaphylaxis was documented at sequential timed intervals, levels of some mediators correlated with delayed deterioration, supporting recommendations for safe observation periods after initial treatment [24].

The guidelines also concur that blood pressure, cardiac rate and function, respiratory status and oxygenation should be monitored clinically at frequent intervals (every 1-5 minutes), or if possible, continuously [2-4]. Lack of universal availability of continuous electronic monitoring and pulse oximetry remains a concern.

### Post-discharge management of patients treated for acute anaphylaxis

Collaborating organizations’ guidelines concur that management of anaphylaxis does not end with treatment of the anaphylactic episode [2-4] and that post-discharge management should include follow-up with a physician, preferably an allergy/immunology specialist [2,4,84-124] (Table 5).

The WAO Guidelines state that if epinephrine auto-injectors are not available, alternative, although not preferred, recommendations for epinephrine injection need to be provided (for details, please see the subsequent section on Post-Discharge Management on page 12 of this paper) [2,6,7]. They also note the importance of anaphylaxis emergency action plans and medical identification (ID) stating anaphylaxis triggers and co-morbid diseases. The AAAAI/ACAAI Guidelines recommend prescribing more than one epinephrine auto-injector because more than one dose of epinephrine is needed in about 20% of anaphylactic episodes; additionally, they recommend action plans and medical ID [3]. The EAACI Guidelines include comprehensive information about discharge management, for example, providing a discharge letter for the family doctor, as well as providing a discharge sheet for patients that contains information about epinephrine auto-injector use, allergen avoidance, and how to contact patient support groups. They also include detailed information about management plans, evidence-based absolute indications for prescription of at least one auto-injector, and suggested indications for prescription of a second auto-injector. They suggest strategies for training patients at risk and caregivers of patients at risk. Additionally, they list gaps in the evidence supporting recommendations for long-term management of anaphylaxis [4] (Table 5).

Post-discharge management of patients at risk for anaphylaxis recurrences in the community is an active area of research [84-98]. This focuses on patient and caregiver failure to carry auto-injectors and use auto-injectors for anaphylaxis [87,88], patient, caregiver, and physician experiences in using auto-injectors [89-92], auto-injector redesign [93,94] and education and support of patients at risk [95-97], an area in which additional high-quality studies are needed [98]. Although improved rates of filling epinephrine prescriptions after discharge from some emergency departments are reported [85,86], for many patients lack of affordable auto-injectors remains a barrier to use [7].

### Investigations to confirm anaphylaxis triggers

The guidelines concur that triggers should be confirmed by re-taking the history of the anaphylactic episode and using this as a guide to selection of allergens for skin prick tests, measurement of allergen-specific IgE levels in serum, and additional investigations as needed [2-4,13-15,22,53,99-106]. Intradermal tests are helpful in investigation of anaphylaxis induced by insect venoms or drugs such as beta-lactam antibiotics [103-106]. Negative skin tests and absent or undetectable allergen-specific IgE levels have a high negative predictive value; however, positive tests have a lower positive predictive value because allergen sensitization without symptoms is widespread in the general population. Ideally, tests to assess sensitization to allergens should be interpreted by an allergy/immunology specialist [2-4,13-15,22,53,64,99-106] (Table 5). Medically-supervised incremental allergen challenge tests, indicated in some patients with food or drug allergy, should be conducted only by experienced healthcare professionals in settings where anaphylaxis can be treated promptly [2-4,13,15,22,53,104,105].

None of the guidelines emphasize standardization of tests and challenges; perhaps because international consensus documents on standardization were only published in 2012 and 2013 [2-4,100,102].

### Prevention of anaphylaxis recurrences

The guidelines concur about prevention of anaphylaxis recurrences by avoidance of confirmed allergens, including hidden or cross-reacting allergens [2-4,13-15,22,53,64,99,104,105],[107-111]. Vigilant avoidance prevents anaphylaxis recurrence from culprit allergens [107,108]; however, it can be time-consuming, frustrating, difficult to sustain in daily life, and associated with impaired quality-of-life; including bullying of food-allergic children [109-111].

The guidelines concur in their recommendation for immune modulation to prevent recurrences of anaphylactic episodes from stinging insect venom [2-4,14,112-116] and drugs [2-4,15,117]. The WAO Guidelines and EAACI Guidelines describe oral immunotherapy (OIT) to prevent recurrences of food-induced anaphylaxis, but concur that this approach is not yet ready for general use [2,4,13,22,119] (Table 5).

For prevention of recurrence of stinging insect venom-induced anaphylaxis, a 3-5 year course of subcutaneous immunotherapy with the relevant standardized specific venom(s) leads to long-lasting protection in most patients [2-4,14,112-115]; lifelong venom immunotherapy (VIT) is recommended in patients with mastocytosis [116].

For prevention of recurrent anaphylaxis from a drug such as an antibiotic or NSAID, or a biologic agent, when no safe substitute is available, desensitization conducted by experienced healthcare professionals using a published protocol is safe and effective for one uninterrupted course of treatment [2-4,15,104,105,117].

In carefully selected patients with symptoms after ingestion of milk, egg, peanut, or other highly allergenic food, RCT of OIT confirm that clinical desensitization can be achieved in most patients; however, sustained unresponsiveness after stopping treatment is more difficult to achieve, and adverse events, including anaphylaxis, occur [99,119,120]. OIT safety can be improved with omalizumab pre-treatment and co-treatment [99,121]. Sublingual immunotherapy to prevent food-induced anaphylaxis, although less effective than OIT, is associated with fewer adverse events [99,120].

The guidelines differ in their emphasis on pharmacologic prophylaxis of anaphylaxis from various triggers. They all describe pharmacologic interventions to prevent anaphylaxis to radiocontrast media [2-4,64,122]. The WAO and the AAAAI/ACAAI Guidelines recommend pharmacologic prophylaxis in selected patients with idiopathic anaphylaxis [2,3]. The EAACI Guidelines provide information about pretreatment with epinephrine to prevent anaphylaxis to snake anti-venom [4,123]. Surprisingly, no guidelines provide information about pharmacological prophylaxis of anaphylaxis from subcutaneous allergen immunotherapy, although H_1_-antihistamine pre-treatment before venom injections during VIT reduces systemic adverse events and has a beneficial immune-modifying effect [124] (Table 5).

### Anaphylaxis education

The guidelines differ in their emphasis on anaphylaxis education for patients and caregivers. The WAO Guidelines outline the principles of anaphylaxis education [2]. The AAAAI/ACAAI Guidelines discuss anaphylaxis education in the context of some specific triggers [3]. The EAACI Guidelines provide comprehensive information about anaphylaxis education, including information about long-term management, recommendations for training (with description of barriers to and facilitators of implementation), audit criteria, and resource implications [4] (Table 5).

None of the guidelines describe anaphylaxis education for personnel working in child care, schools, colleges, universities, summer camps, and sports facilities, or the hospitality or airline industries; however, a forthcoming EAACI publication addresses anaphylaxis education in the community-at-large (Muraro A, personal communication).

### Follow-up with a physician

All the guidelines address the issue of follow-up with a physician [2-4], if possible with an allergy/immunology specialist. The WAO Guidelines recommend follow-up yearly for review of prevention of recurrence, epinephrine auto-injector use, and optimizing control of relevant co-morbid diseases such as asthma [2]. The AAAAI/ACAAI Guidelines discuss specific aspects of follow-up in association with some of the major triggers [3]. In addition to physician follow-up, the WAO and EAACI Guidelines note the importance of follow-up with a dietician, if relevant, and the EAACI Guidelines also suggest follow-up with a psychologist, if relevant [4] (Table 5).

## Unmet needs in anaphylaxis

Unmet needs in anaphylaxis in high-, mid-, and low-resource countries are described in Tables 6, 7, 8, and 9.

**Table 6:** Essential information on anaphylaxis: summary of unmet needs

	**High-resource countries**^**1**^	**Limited-resource countries**^**2**^
**Definition of anaphylaxis, eg. serious, life-threatening generalized allergic or hypersensitivity reaction**	need ↑ awareness of a current anaphylaxis definition for clinical use	need ↑ awareness of a current anaphylaxis definition for clinical use
**Epidemiology**	need integration of the clinical criteria for diagnosis of anaphylaxis with ICD-9 and ICD-10 codes; need more reliable prevalence estimates in healthcare settings and in the general population; and need more reliable prevalence estimates of mortality rates from different triggers in different patient populations	need concurrent epidemiologic studies of anaphylaxis using similar methods in different countries in order to obtain reliable prevalence estimates in the general population
**Patient risk factors and co-factors relevant to anaphylaxis**	need ↑ awareness of patient risk factors for severe or fatal anaphylaxis, eg. asthma, CVD and MCAD; and need ↑ awareness of the role of co-factors such as exercise, ethanol, NSAIDs, emotional stress, acute infection, perimenstrual status	need baseline information about the prevalence of asthma, CVD, and MCAD so their relationship with anaphylaxis can be ascertained; need ↑ awareness of potential patient risk factors and co-factors; insights might be obtained from studies of fatal episodes^3^
**Underlying mechanisms**	need greater understanding of IgE-dependent, IgE-independent, and non-immunologic mechanisms (direct mast cell activation)	need greater understanding of IgE-dependent, IgE-independent, and non-immunologic mechanisms
**Triggers (causes, elicitors, or inducers)**	need improved standardization of allergens, a standardized mechanism for reporting novel triggers, and continued efforts to standardize protocols for skin tests and challenge tests	need more comprehensive information about newly-discovered allergen triggers, allergens in some geographic areas, and certain groups of allergens, eg. reptile venoms and helminths

^1^Within high-resource countries, limited-resource areas can be found in inner cities, some rural areas, many public venues, and situations such as anaphylaxis on airplanes.

^2^In this Table, “limited-resource countries” include mid- and low-resource countries.

^3^In some limited-resource countries, fewer than 5% of deaths are certified with regard to cause.

CVD, cardiovascular disease; ICD, International Classification of Disease; MCAD; mast cell activation disorders; NSAIDs, non-steroidal anti-inflammatory drugs.

**Table 7:** Diagnosis of anaphylaxis: summary of unmet needs

	**High-resource countries**^**1**^	**Limited-resource countries**^**2**^
**Symptoms and signs (typically with onset in minutes to a few hours after exposure; multi-system; rapid progression)**	need improved recognition and diagnosis of anaphylaxis and its sudden onset in relationship to exposure to a trigger (the context) among all healthcare professionals (including EMS personnel), patients, caregivers and the public	need improved recognition and diagnosis of anaphylaxis and its sudden onset in relationship to exposure to a trigger (the context) among all healthcare professionals, patients, caregivers, and the public
**Clinical diagnosis of anaphylaxis**	need to operationalize the clinical criteria for the diagnosis of anaphylaxis by healthcare professionals; need improved coding for anaphylaxis and training of coders	need to operationalize the clinical criteria for the diagnosis of anaphylaxis; need ↑ availability of basic equipment and supplies to aid in recognition, eg. pulse oximetry and sphygmomanometers with arm cuffs of various sizes
**Laboratory tests to confirm the clinical diagnosis eg. serum total tryptase measurement**^**3**^	need ↑ awareness of optimal timing of tests, and lack of test specificity (eg. ↑ tryptase in some patients with MI); need ↑ awareness that current lab tests are not useful at the time of patient presentation; need ongoing development of tests for biologic markers	need awareness that inability to measure tryptase levels (which is unlikely to be a priority for change due to high cost) is ***not*** a barrier to prompt diagnosis of anaphylaxis, which depends on recognition of symptoms and signs
**Differential diagnosis**	need ↑ awareness that among the >40 entities in the differential diagnosis, acute asthma, acute urticaria, and panic or anxiety attacks are most common	need awareness that in some countries, pneumonia and sepsis are the most likely diagnoses in patients with sudden-onset respiratory distress and sudden-onset hypotension/ distributive shock, respectively
**Diagnosis of anaphylaxis in special populations**	need improved prompt recognition of anaphylaxis in infancy, adolescence, pregnancy, and advanced age	need improved prompt recognition of anaphylaxis in infancy, adolescence, pregnancy, and advanced age

^1^Within high-resource countries, limited-resource areas can be found in inner cities, some rural areas, many public venues, and situations such as anaphylaxis on airplanes.

^2^In this Table, “limited-resource countries” include mid- and low-resource countries.

^3^The tryptase assay is standardized worldwide; in contrast, histamine assays are not standardized and are less practical for clinical use because of the need to refrigerate specimens.

EMS, emergency medical services; MI, myocardial infarction.

**Table 8:** Treatment of anaphylaxis in healthcare settings: summary of unmet needs

	**High-resource countries**^**1**^	**Limited-resource countries**^**2**^
**Prompt initial treatment of anaphylaxis**	need to encourage the “be prepared” approach: have a protocol, inject epinephrine promptly IM in mid-outer thigh, call for help, and position the patient appropriately; need to reduce the fear factor associated with epinephrine use by stressing the good benefit/harm ratio of prompt IM epinephrine compared with IV epinephrine	need to develop simple protocols, and focus on essentials: inject epinephrine promptly IM in mid-outer thigh, call for help, position the patient appropriately; need to ensure the availability of epinephrine as an essential drug in 1 mg/mL ampules in all healthcare facilities, and improve availability of low-cost EAIs (or if EAIs are unaffordable, factory-sealed syringes prefilled with epinephrine 1 mg/mL)
**Initial treatment (cont.)**	need ↑ awareness that H_1_-antihistamines, H_2_-antihistamines, and glucocorticoids are not ***initial*** medications of choice in anaphylaxis and should not be used as monotherapy	need ↑ availability of supplemental oxygen and IV fluids; where oxygen cylinders are not available, oxygen concentrators can be useful; lack of availability of supplemental oxygen and IV fluids is more critical than lack of second-line medications such as antihistamines and glucocorticoids, which are not essential for survival
**Management of anaphylaxis refractory to initial treatment**	need to identify hospitals where patients with refractory anaphylaxis can receive skilled ventilatory and inotropic support from experienced, well-equipped personnel, and to list the contact information for these facilities on the anaphylaxis protocol	need ↑ availability of supplemental oxygen and IV fluids such as 0.9% saline; need regular reassessment of availability of epinephrine, supplemental oxygen and IV fluids, supplies and equipment in hospitals
**Observation and monitoring in healthcare settings**	need ↑ availability of continuous electronic monitoring of cardiac rate, function, and blood pressure, and of pulse oximetry	need ↑ availability of basic supplies and equipment for monitoring in many hospitals; and improved availability of continuous electronic monitoring of cardiac rate, function, and blood pressure, and pulse oximetry in teaching hospitals

^1^Within high-resource countries, limited-resource areas can be found in inner cities, some rural areas, many public venues, and situations such as anaphylaxis on airplanes.

^2^In this Table, “limited-resource countries” include mid- and low-resource countries.

EAIs, epinephrine auto-injectors; IM, intramuscular; IV, intravenous.

**Table 9:** Treatment of anaphylaxis in community settings: summary of unmet needs

	**High-resource countries**^**1**^	**Limited-resource countries**^**2**^
**Discharge management of patients treated for anaphylaxis**	need ↑ public awareness of the importance of prompt anaphylaxis recognition and first-aid treatment for patients with anaphylaxis in the community; need ↑ availability of low-cost EAIs and of “stock” epinephrine^3^ in schools, shopping malls, etc.; need a wider range of epinephrine doses in auto-injectors, eg. 0.1 mg and 0.5 mg	need ↑ availability of low-cost EAIs or even factory-sealed prefilled epinephrine syringes; need ↑ awareness of alternative but not preferred options (epinephrine 1 mg/1 mL ampules and 1 mL syringes, and unsealed syringes prefilled by healthcare professionals); need more information about epinephrine shelf-life in extreme climates
**Investigations to confirm anaphylaxis triggers**	need improved standardization of allergens and of test and challenge protocols; need ↑ awareness that allergen sensitization is far more common than clinical symptoms; and that tests for sensitization must be selected and interpreted based on the history of the anaphylactic episode	need ↑ awareness that if sterile needles are available, allergen skin tests can be performed by skin prick or prick-prick testing with relevant foods, or skin testing with IV formulations of medications
**Prevention of anaphylaxis recurrences**	need improved public policies with regard to food labeling, improved school policies for anaphylaxis prevention and treatment, and improved access to specialists, including those who can document sensitization to novel triggers	need improved training of healthcare professionals to identify anaphylaxis triggers, symptoms, and signs; need ↑ availability of tests to confirm sensitization; (in their absence, trigger avoidance is based on the history); need ↑ availability of venom immunotherapy and desensitization to drugs
**Anaphylaxis education**	need ↑ availability of personalized anaphylaxis education by trained healthcare professionals and development of personalized emergency action plans that focus on recognition of symptoms and signs, implementation of the plan, prompt use of EAI, and wearing medical ID	need ↑ awareness of anaphylaxis, improved training of healthcare professionals, and development of action plans to aid in recognition of anaphylaxis symptoms and signs; need improved availability of EAIs
**Follow-up**	need ↑ awareness of importance of follow-up with an allergist/ immunologist to provide training in anaphylaxis recognition, EAI use, allergen avoidance; and when indicated, immune modulation, eg. VIT	need ↑ awareness of the importance of follow-up after an acute anaphylactic episode; availability of follow-up will depend on local conditions

^1^Within high-resource countries, limited-resource areas can be found in inner cities, some rural areas, many public venues, and situations such as anaphylaxis on airplanes.

^2^In this Table, “limited-resource countries” include mid- and low-resource countries.

^3^Rationale: preventable deaths, especially in children, teenagers, and young adults occur in these venues; this issue is also listed in the research agenda (Table 11) because of the need to gather additional data.

EAIs, epinephrine auto-injectors; ID, identification; VIT, venom immunotherapy.

### Definition of anaphylaxis

In all countries, increased awareness of anaphylaxis as a serious, life-threatening, generalized or systemic, allergic or hypersensitivity reaction with sudden onset (minutes to a few hours) is needed among healthcare professionals, patients, caregivers, and the public (Table 6).

### Epidemiology

For epidemiologic purposes, the validated clinical criteria for anaphylaxis diagnosis are helpful for informing International Classification of Disease (ICD)-9 and ICD-10 codes and facilitating reliable estimates of anaphylaxis prevalence in healthcare settings [27-33] and to a lesser extent in the general population [34,35].

In all countries, epidemiological and health services research can serve as a baseline for quality improvement, prioritization of anaphylaxis programs, and eventual reduction in morbidity and mortality. However, until diagnosis of anaphylaxis as such by healthcare professionals improves and recognition by patients, caregivers, and the public improves, it will remain difficult to obtain reliable epidemiologic information about anaphylaxis and its prevalence will remain under-estimated (Table 6).

At post-mortem, too, anaphylaxis can be under-diagnosed [36]; for example, when signs of anaphylaxis are absent and recognition is based only on circumstantial evidence and exclusion of other diseases [37]. Nevertheless, anaphylaxis fatality studies can sometimes provide unique information about triggers, presenting symptoms and signs, time course, and associated co-morbidities in a specific region or country.

### Patient-specific risk factors and co-factors relevant to anaphylaxis

In all countries, improved recognition of patient vulnerability to anaphylaxis is needed as related to age, physiologic state (pregnancy), concomitant diseases, concurrent medications, and amplifying co-factors. In some countries, the prevalence of co-morbid diseases such as asthma or CVD might itself not be known (Table 6).

### Underlying mechanisms and anaphylaxis triggers

Positive collaborations among physicians in academic centers and national allergy/immunology professional organizations in high-resource countries with those in mid- or low-resource countries are expanding our global knowledge of anaphylaxis mechanisms and triggers [36,37,56,57,123]. Despite this, in some countries, little information about anaphylaxis triggers, even the taxonomy of indigenous food plants and stinging insects, is available. Where potential triggers remain unidentified, lack of context for recognition of anaphylaxis can delay diagnosis and treatment (Table 6).

### Clinical diagnosis of anaphylaxis

In all countries, improved training of healthcare professionals to recognize and treat anaphylaxis is needed, and the validated clinical criteria for anaphylaxis diagnosis need to be operationalized in order to optimize their usefulness. Where resources are limited, there can be inconsistent availability of basic services such as electricity and of equipment and supplies that aid in anaphylaxis diagnosis; for example, pulse oximeters to document oxygenation and sphygmomanometers and arm cuffs of various sizes to document blood pressure [125-130].

In such situations, assessment of hypoxemia is based on clinical indicators such as central cyanosis, nasal flaring, inability to speak or drink, grunting, lethargy, severe chest retractions, respiratory rate of more than 70 breaths/minute, and head-nodding; a capillary refill time of 2 seconds or less is documented to reflect a superior vena caval oxygen saturation of greater than 70%. Assessment of hypotension and distributive shock is based on clinical indicators such as weak or non-palpable peripheral pulses [125-127] (Table 7).

In all countries, in the differential diagnosis of anaphylaxis, healthcare professionals should be aware of common considerations such as acute asthma, acute urticaria, and panic or anxiety attacks. In some countries, pneumonia is the most common consideration in patients presenting with acute-onset respiratory distress and hypoxemia, and sepsis is the most common consideration in those presenting with acute-onset hypotension or shock [2-4,125,126] (Table 7).

### Prompt initial treatment of anaphylaxis and monitoring

In all countries, at the onset of an anaphylactic episode, it can be impossible to predict the rate of escalation or resolution of symptoms. Patients can present with deceptively mild symptoms such as hives, cough, or dizziness that rapidly increase in severity and culminate in fatality within minutes. Regardless of available resources, an important message to healthcare professionals, patients, caregivers, and the public is to recognize anaphylaxis promptly and as soon as it is recognized, inject life-saving epinephrine in order to maximize the likelihood of survival [2-4].

Box: The essentials of prompt initial anaphylaxis treatment

- have an easy-to-follow and well-rehearsed protocol - remove exposure to the trigger, if relevant - inject epinephrine promptly intramuscularly in the mid-outer thigh - call for help (resuscitation team in hospital or emergency medical services in community, if available) - position the patient supine (or semi-reclining in a position of comfort if dyspneic or vomiting) and elevate the lower extremities ***If indicated at any time,*** - provide supplemental oxygen - initiate IV fluid resuscitation with 0.9% saline - perform cardiopulmonary resuscitation - monitor BP, cardiac rate and function, and oxygen saturation

See Table 8 for details.

Where resources are limited, supplemental oxygen can be provided by oxygen concentrators instead of oxygen cylinders, and nasal prongs or nasopharyngeal catheters can be substituted for oxygen masks [128,129]; however, in many hospitals, lack of availability of pulse oximetry for detecting hypoxemia and guiding oxygen therapy remains a critical concern [127]. Despite best efforts, treatment of anaphylaxis can, in some patients, be compromised by co-morbidities such as anemia and reduced ability to achieve adequate oxygenation, or severe malnutrition and reduced ability to tolerate IV fluid resuscitation [125,126] (Table 8).

### Management of anaphylaxis refractory to initial treatment

Even in high-resource countries, optimal treatment of refractory anaphylaxis is not available universally; for example, in remote, inaccessible, or impoverished areas or in specific situations such as anaphylaxis on airplanes. In limited-resource situations, lack of availability of basic essentials such as epinephrine, supplemental oxygen and IV fluid resuscitation is more critical than lack of second-line medications such as antihistamines and glucocorticoids. Lack of availability of advanced life-support management can be a major barrier to survival [72-76,128] (Table 8). In any limited-resource situation, resuscitation efforts prolonged over hours using a hand-held bag valve mask (manual resuscitator) are often successful in anaphylaxis [2] (Table 8).

In mid- and low-resource countries, striving to ensure more consistent availability of medications, supplies, and equipment for anaphylaxis treatment is an important goal [2,5-7]. The World Health Organization has developed a tool kit containing evidence-based guidelines and a framework for quality improvement in the hospital care of critically ill children in such environments [126], where despite many obstacles, improvements can be documented [130].

### Post-discharge management

In high-resource countries, there is an increased focus on post-discharge management after successful treatment of anaphylaxis. Where resources are limited, post-discharge management is severely compromised by lack of availability of affordable auto-injectors or factory-sealed prefilled syringes containing epinephrine [2,6,7]. There are two alternative, although not preferred, options for epinephrine self-administration. First, a 1 mL ampule of epinephrine and a 1 mL syringe can be provided; however, in a medical emergency, patients without medical training find it difficult to draw up a dose accurately and expel air from the syringe without losing the epinephrine. Second, an unsealed prefilled syringe containing the correct dose for the patient can be drawn up in advance by his/her physician; however, all or part of the dose can be lost, and epinephrine is stable for only 3-4 months in an unsealed syringe [2,6,7].

Where resources are limited, local conditions typically determine the availability of follow-up with a healthcare professional and prevention of recurrent anaphylactic episodes is often compromised by lack of availability of physicians, tests to confirm triggers, and immune modulation (Table 9).

## International research agenda for anaphylaxis

Anaphylaxis research has been hindered in the past by the perception that the disease is rare, absence of a universally accepted definition for clinical use, and lack of validated criteria for anaphylaxis diagnosis suitable for use in clinical and epidemiologic studies. Progress in these areas is giving momentum to basic, translational, and clinical anaphylaxis research.

ICON: Anaphylaxis proposes a comprehensive international research agenda for anaphylaxis (Tables 10 and 11) that extends and amplifies the anaphylaxis research agendas published independently by WAO and EAACI [2,4]. The ICON: Anaphylaxis research agenda is based in part on identification of areas where little or no high quality evidence is available to support the recommendations for anaphylaxis diagnosis, treatment, and prevention made in anaphylaxis guidelines and other publications.

**Table 10:** **International research agenda for anaphylaxis**^**1,2**^


**Epidemiology, Patient Risk Factors, Mechanisms, Triggers, and Diagnosis**
**Epidemiology of anaphylaxis**
Prospective studies of:
- global incidence and prevalence of anaphylaxis in general populations in different countries, in order to obtain reliable population estimates; ideally, concurrent studies will be performed
- anaphylaxis from all triggers, and from specific triggers including foods, stinging insect and other venoms, drugs, etc.
- anaphylaxis in different populations: infants, children, teenagers, pregnant women, the elderly, and patients with co-morbidities such as asthma, cardiovascular disease, and mast cell activation disorders
- the natural history of anaphylaxis based on well-designed longitudinal population-based investigations
**Patient risk factors for anaphylaxis**
Genotypes, phenotypes and endotypes of patients with anaphylaxis
Development of instruments to quantify patient-specific risk factors, ascertain their relative importance, and predict future anaphylactic episodes
Biologic markers for identification of patients at risk
Prospective studies of the relationship between food-induced anaphylaxis and asthma, in order to ascertain the relationship of anaphylaxis severity and asthma control
Prospective studies of the relationship between food, insect venom, and drug-induced anaphylaxis and cardiovascular disease
Prospective studies of the relationship between anaphylaxis and mast cell activation disorders
Prospective studies of idiopathic anaphylaxis in patients of all ages
**Anaphylaxis mechanisms**
Further elucidation of mechanisms underlying anaphylaxis, including studies to improve understanding of molecular mechanisms
Studies of IgG-mediated anaphylaxis in humans
Additional studies of agents that can induce anaphylaxis through more than one mechanism, eg. radiocontrast media, biological agents such as infliximab, etc.
Further elucidation of the role of amplifying co-factors in anaphylaxis
**Triggers (causes, elicitors, inducers) of anaphylaxis**
Prospective studies of trends in triggers, to identify those that are becoming more (or less) common in different patient populations and in different global regions
Additional investigations of food cross-reactivities
Improved methods to detect hidden food allergens
Improved tests to confirm sensitization to anaphylaxis triggers that are uncommon in many countries, but relatively common in others; for example:
- foods such as buckwheat, silkworm pupa, bird's nest soup, chickpea, flour mites, maize, manioc
- stings and bites, eg. ants, caterpillars, jellyfish, lizards, scorpions, snakes
**Diagnosis of anaphylaxis**
Development of operationalized clinical criteria for the diagnosis of anaphylaxis
Validation of these operationalized clinical criteria for use in additional healthcare settings, in community settings, and in different countries
Development and validation of an algorithm for diagnosing anaphylaxis based on clinical criteria
Identification of additional biologic markers for identification of anaphylaxis
Further development of tests for biologic markers that might be useful for confirming the diagnosis of anaphylaxis at the time the patient presents
Development of protocols and algorithms to improve post-mortem identification of anaphylaxis as a cause of death

^1^Basic, clinical and applied sciences.

^2^This Table extends and amplifies the agendas for anaphylaxis research published independently by WAO and by EAACI.

**Table 11:** **International research agenda for anaphylaxis**^**1,2**^


**Management of Anaphylaxis in Healthcare and Community Settings, Risk Assessment and Reduction, and Education**
**Treatment in healthcare settings**
Epinephrine pharmacokinetic and pharmacodynamic studies in patients with different body mass indices
Additional comprehensive studies of epinephrine absorption after different routes of administration, including auto-injectors
Additional observational investigations of the safety of a first-aid dose of epinephrine (0.3 mg intramuscularly) in patients with cardiovascular disease
Multicenter prospective randomized controlled trials to define the role of other pharmacologic interventions in anaphylaxis - examples include H_1_-antihistamines, H_2_-antihistamines, glucocorticoids, and glucagon
**Management in community settings**
Additional comparative studies of different epinephrine auto-injectors
- preference to carry, preference to use, and rate of occurrence of unintentional injections and injuries
Evaluation of the role of “stock” or “unassigned” epinephrine auto-injectors in public places, eg. schools, shopping malls
Further assessment of costs of epinephrine auto-injectors and their cost-effectiveness
Further evaluation of other routes of epinephrine administration, eg. sublingual, inhaled, intranasal
Prospective validation studies of anaphylaxis emergency action plans
Comparison of different anaphylaxis emergency action plans
Assessment of effectiveness of anaphylaxis emergency action plans
Assessment of school plans for anaphylaxis
**Risk assessment in anaphylaxis**
Further standardization of allergens, allergen skin test protocols, and allergen challenge protocols to facilitate comparisons among centers
Further prospective studies of optimal timing of allergen skin tests after anaphylaxis to foods, venoms, drugs, and other allergens
Further development of in vitro tests such as component-resolved diagnostics and basophil activation tests to help distinguish asymptomatic sensitization from clinical risk
Development of new non-invasive tests to assess sensitization versus risk of clinical reactivity to drugs
**Long-term risk reduction in anaphylaxis**
Further prospective investigations of efficacy and safety of oral, sublingual, and epicutaneous immunotherapy to prevent recurrence of food-induced anaphylaxis and achieve immunologic tolerance
Further studies of the efficacy and safety of omalizumab pre-treatment and co-treatment with allergen immunotherapy
Studies of allergen immunotherapy to prevent anaphylaxis recurrences from less well-studied allergens, eg. natural rubber latex
Additional studies of immunotherapy to prevent recurrence of venom-induced anaphylaxis and immune modulation to prevent recurrence of drug-induced anaphylaxis
Additional prospective investigations of pharmacologic prophylaxis of iatrogenic anaphylaxis from radiocontrast media, biologic agents, snake anti-venom, allergen immunotherapy, etc.
Prospective investigations of the utility and cost-effectiveness of providing epinephrine auto-injectors to all patients receiving subcutaneous allergen immunotherapy with aeroallergens or venoms
**Anaphylaxis education**
Studies of methods to increase anaphylaxis awareness among patients, caregivers, and the public
Evaluation of educational programs for all physicians, including emergency medicine and primary care physicians
Evaluation of educational programs for other healthcare personnel, including nurses and paramedics
Evaluation of educational programs for patients at risk and caregivers
Studies of the unique needs of adolescents at risk for anaphylaxis recurrence in community settings and how best to communicate effectively with them
Evaluation of educational programs for the public
Studies of resistance to change and how to facilitate change
**Other**
Studies on anaphylaxis guidelines implementation
Studies on development of anaphylaxis pathways

^1^Basic, clinical and applied sciences.

^2^This Table extends and amplifies the agendas for anaphylaxis research published independently by WAO and by EAACI.

Research tasks awaiting prioritization, as listed in Table 10, include operationalizing the clinical criteria for diagnosis and additional studies of epidemiology, patient risk factors, mechanisms, and triggers. Research tasks awaiting prioritization, as listed in Table 11, include further RCT of interventions, risk assessment, long-term risk reduction, and anaphylaxis education, as well as studies on anaphylaxis guidelines implementation and development of anaphylaxis pathways.

The ICON: Anaphylaxis research agenda will require regular updating and might take decades to complete, depending on the collaborations initiated and the financial support available. Prioritization of research questions is recommended. Initially this should involve identification of questions that are feasible to answer in the short-to-medium term, ideally guided by a formal consensus-building process involving basic scientists, methodologists, and clinician scientists.

Global collaborative efforts to date are improving the diagnosis and treatment of anaphylaxis [36,37,56,57,123]. They have identified the importance of using the validated clinical criteria to inform ICD-10 codes for improved accuracy of anaphylaxis identification at autopsy [36], and found differences between culprit allergens and circumstances of death from anaphylaxis in different countries [37]. They have also elucidated the role of novel anaphylaxis triggers, for example, flour mites [56] and short-chain galacto-oligosaccharides [57], and confirmed in a RCT that epinephrine pre-treatment reduced anaphylaxis to anti-snake venom by 43% and was superior to H_1_-antihistamine and glucocorticoid pre-treatment [123]. Global collaboration among investigators needs to be facilitated in order to accelerate future advances.

## Summary

ICON: Anaphylaxis presents a harmonized approach to anaphylaxis diagnosis, treatment, and prevention based on the alignment found in the collaborating organizations’ principal anaphylaxis guidelines. It documents consensus in the critically important areas of clinical diagnosis, treatment and prevention of anaphylaxis recurrences and, further, documents unmet needs in these areas. It recommends increasing the awareness of anaphylaxis, continuing to strengthen the evidence supporting recommendations for management and prevention, and improving dissemination and implementation of anaphylaxis guidelines. It proposes a comprehensive international anaphylaxis research agenda and calls for facilitation of increased collaborations among investigators in high-, mid- and low-resource countries. ICON: Anaphylaxis is a unique resource for physicians, other healthcare professionals, academics, policy-makers, patients, caregivers, and the public worldwide.

## Abbreviations

AAAAI: American Academy of Allergy Asthma and Immunology; ACAAI: American College of Allergy, Asthma and Immunology; ACE: Angiotensin-converting enzyme; BP: Blood pressure; CVD: Cardiovascular disease; EAACI: European Academy of Allergy and Clinical Immunology; EAI: Epinephrine auto-injectors; EMS: Emergency medical services; iCAALL: International Collaboration in Asthma, Allergy and Immunology; ICD: International Classification of Disease; ICON: Anaphylaxis – international consensus on anaphylaxis; ID: Identification; IM: Intramuscular; IV: Intravenous; MCAD: Mast cell activation disorder; MI: Myocardial infarction; NSAID: Non-steroidal anti-inflammatory drugs; OIT: Oral immunotherapy; RCT: Randomized controlled trials; RCM: Radiocontrast media; VIT: Venom immunotherapy; WAO: World Allergy Organization.

## Competing interests

F. Estelle R. Simons: member, Medical Advisory Boards of ALK, Mylan, and Sanofi. Ledit Ardusso: no competing interests. Maria Beatrice Bilo: ALK, Meda. Victoria Cardona: has received fees as an advisor and speaker for ALK. Motohiro Ebisawa: no competing interests. Yehia El-Gamal: no competing interests. Phil Lieberman: member of the medical advisory boards of, and has been a consultant to, Mylan and Sanofi-Aventis; speaker for Mylan. Richard Lockey: no competing interests. Antonella Muraro: has served as advisor for, and has received speaker fees from, Meda. Graham Roberts: member, Medical Advisory Board for ALK-Abello. Mario Sanchez-Borges: World Allergy Organization Executive Committee (President-Elect). Aziz Sheikh: has received honoraria for consultancy and/or research from ThermoFisher, ALK, Meda, and Allergy Therapeutics. Lynette Shek: no competing interests. Dana Wallace: advisor/consultant for Mylan and Sanofi. Margitta Worm: has received honoraria for consultation and lectures from Meda, ALK, and Allergopharma.

## Authors’ contributions

FERS led the development of the document and prepared the initial, interim, and final drafts. All authors contributed to the content of the document. All authors reviewed and approved the final document. Please see the “Methods” section on page 10 of this publication for details of the development process.
